# Marking the differences in motoneurons

**DOI:** 10.7554/eLife.36832

**Published:** 2018-04-27

**Authors:** Simon A Sharples, Patrick J Whelan

**Affiliations:** 1 Hotchkiss Brain InstituteUniversity of CalgaryCalgaryCanada; 2 Department of NeuroscienceUniversity of CalgaryCalgaryCanada; 3 Department of Comparative Biology and Experimental Medicine, Faculty of Veterinary MedicineUniversity of CalgaryCalgaryCanada

**Keywords:** electrophysiology, in vivo intracellular recordings, motor neuron, motoneuron, firing properties, Amyotrophic lateral sclerosis, Mouse

## Abstract

A subgroup of the neurons that control muscles becomes less excitable shortly before the symptoms of ALS develop.

**Related research article** Martinez-Silva ML, Imhoff-Manuel RD, Sharma A, Heckman CJ, Shneider NA, Roselli F, Zytnicki D, Manuel M. 2018. Hypoexcitability precedes denervation in the large fast-contracting motor units in two unrelated mouse models of ALS. *eLife* **7**:e30955. doi: 10.7554/eLife.30955

The world's attention was focused on amyotrophic lateral sclerosis (ALS) recently with the death of Stephen Hawking. The famous physicist had a rare form of slowly progressing ALS that resulted in a gradual loss of motor function. Perhaps the experience of the disease is best conveyed by Hawking himself: “I have lived with the prospect of an early death for the last 49 years. I’m not afraid of death, but I’m in no hurry to die. I have so much I want to do first”.

In most cases, ALS progresses quickly, with an average lifespan of 2–5 years following diagnosis. It is a disease that affects the motoneurons that control muscles, but it is curiously selective. Some motoneurons are more vulnerable than others, and ALS researchers have been working to uncover the reasons for this in the hope of identifying ways to protect these cells. Now, in eLife, Marin Manuel and colleagues – including Maria de Lourdes Martinez-Silva of CNRS/Université Paris Descartes as first author – report that ‘fast’ and ‘slow’ motoneurons behave differently during the early stages of ALS (Martinez-Silva et al., 2018).

Motoneurons are a diverse population, but all connect with their target muscles across structures called neuromuscular junctions that break down as ALS progresses. Some motoneurons are large and control muscles that produce fast, forceful contractions, while others are small and control muscles that produce weaker but sustained contractions. The fast motoneuron populations are particularly susceptible to ALS due to the high metabolic demands of their greater size.

For a long time, the death of the fast motoneurons was believed to be partly due to them becoming ‘hyperexcitable’, meaning that they fire too easily and too often. This leads to calcium ions accumulating inside the cells, which may trigger cell death. This theory was supported by the finding that a drug called riluzole could increase life expectancy by a few months by blocking the release and reception of excitatory neurotransmitters (Rothstein, 2009). However, the hypothesis that hyperexcitability causes cell death was challenged by a report that it may instead delay the progress of ALS (Saxena et al., 2013).

Molecular and electrical markers have been developed that can identify fast and slow motoneurons in vitro (Leroy et al., 2014), and these markers have been used to demonstrate that slow – but not fast – motoneurons are hyperexcitable during the weeks after birth in a mouse model of ALS. Unfortunately, the markers cannot distinguish fast from slow motoneurons in adulthood, which is when the symptoms of ALS normally emerge.

Martinez-Silva et al. – who are based in Paris, Columbia University, Northwestern University and Ulm University – have now stimulated individual motoneurons in anesthetized mice while simultaneously recording the electrical response from motoneurons and the force generated by muscles (Figure 1A). This approach allows for fast and slow motoneuron subtypes to be identified directly from their different responses in muscle to the stimulation of the motoneurons (Figure 1B). The key finding of these experiments is that the fast motoneurons become less responsive to repetitive stimulation – that is, they become hypoexcitable – shortly before ALS symptoms become apparent in the mice, while the neuromuscular junctions are still intact. However, the slow motoneurons remain unaffected. These results were replicated in two unrelated genetic mouse models of ALS.

**Figure 1.**
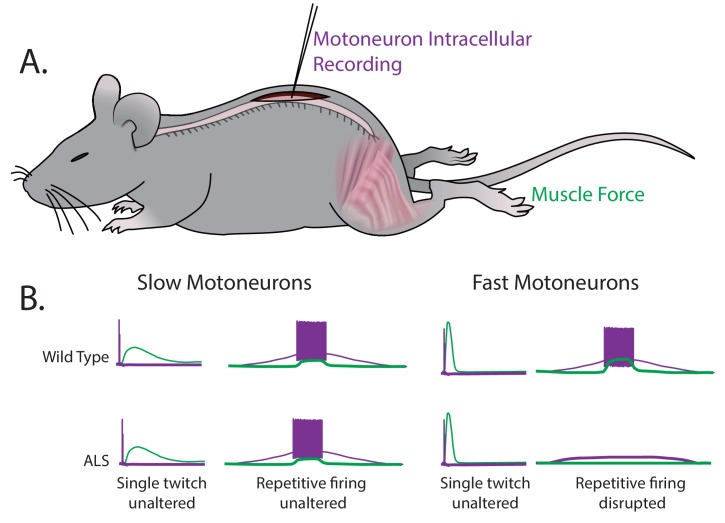
Fast and slow motoneurons are affected differently by the early stages of ALS. (**A**) Martinez-Silva et al. recorded directly from motoneurons innervating fast and slow twitch muscles in anesthetized mice. (**B**) Motoneurons were identified by simultaneously recording the electrical properties inside the cells (purple) and measuring the muscle force (green) in wild type mice (top graphs) and mouse models of ALS (bottom graphs) as the mice approached the age at which ALS symptoms begin (30–60 days after birth). For slow motoneurons the responses of the muscle to a single action potential (first column) or to repeated action potentials (second column) were similar in the wild type mice and the mouse models of ALS. For fast motoneurons the response to a single action potential (third column) was also similar, but fewer of the fast motoneurons in the mouse model of ALS responded to repeated action potentials (fourth column). Data adapted from Martinez-Silva et al., 2018, Figures 1 and 3 (reused under a CC BY 4.0 license).

Previous research had demonstrated hypoexcitability at late stages of ALS using cultured motoneurons derived from humans (Devlin et al., 2015) or in vitro and in vivo preparations (Delestrée et al., 2014), but it was not clear from those studies which population of motoneurons was affected. As well as distinguishing between the excitability changes in different motoneuron subtypes, Martinez-Silva et al. have also confirmed a previous suggestion that chondrolectin is a marker for fast motoneurons (Enjin et al., 2010). Importantly, they were able to use established biomarkers of ALS to show that the more hypoexcitable fast motoneurons are those that are in more advanced stages of the disease.

Armed with this new understanding of ALS progression, we can start to ask additional mechanistic questions, such as why does hyperexcitability protect motoneurons during the early stages of ALS? And what mechanisms drive the transition from hyper to hypoexcitability? Some have argued that hypoexcitability prolongs cell survival by reducing the flow of calcium ions into previously hyperexcitable motoneurons (Delestrée et al., 2014). This remains a possibility because hyperexcitability can largely be accounted for by increases in excitatory signaling onto motoneurons, rather than the intrinsic properties of these cells (Selvaraj et al., 2018).

Finally, does hypoexcitability directly cause neuromuscular junctions to break down? Based on the age-old mantra ‘cells that fire together wire together’, the reduced activity of a hypoexcitable motoneuron could hold back the growth factors that stabilize the neuromuscular junction. Indeed, recent work shows that protecting the integrity of the neuromuscular junction can prolong life, albeit for a short time (Cantor et al., 2018). The issue remains complex since blocking motoneuron firing with tetrodotoxin does not appear to alter how the disease progresses (Carrasco et al., 2012). Whatever the mechanism, it is clear from the work presented by Martinez-Silva et al. that therapeutic interventions for ALS need to be implemented based on the stage of disease progression.
